# Differential analysis of the impact of lesions’ location on clinical and radiological outcomes after the implantation of a novel aragonite-based scaffold to treat knee cartilage defects

**DOI:** 10.1007/s00264-024-06314-1

**Published:** 2024-09-21

**Authors:** Pietro Conte, Giuseppe Anzillotti, Dennis C. Crawford, Vinod Dasa, David C. Flanigan, William E. Nordt, Jason M. Scopp, Robert J. Meislin, Eric J. Strauss, Sabrina M. Strickland, Gennaro Fiorentino, Christian Lattermann

**Affiliations:** 1 Department of Biomedical Sciences, Humanitas University, Via Rita Levi Montalcini 4, Pieve Emanuele, Milan, 20072 Italy; 2 IRCCS Humanitas Research Hospital, via Manzoni 56, Milan, Rozzano, 20089 Italy; 3 OHSU Department of Orthopaedics & Rehabilitation, Center for Health & Healing, Portland, OR USA; 4 Department of Orthopaedics, School of Medicine, Louisiana State University Health Sciences Center, New Orleans, LA USA; 5 Sports Medicine, Department of Orthopaedics, The Ohio State University Wexner Medical Center, Columbus, OH USA; 6 OrthoVirginia, 23229 Richmond, VA USA; 7 Joint Preservation Center, Peninsula Orthopaedic Associates, P.A, Salisbury, MD USA; 8 Department of Orthopedic Surgery, New York University Langone Health, New York, NY USA; 9 Hospital for Special Surgery, Sports Medicine Institute, New York, NY USA; 10 Department of Orthopaedics and Traumatology, Humanitas Gavazzeni, Bergamo, Italy; 11 Department of Orthopaedic Surgery, Center for Cartilage Repair and Sports Medicine, Brigham and Women’s Hospital, Harvard Medical School, 75 Francis Street, 02115 Boston, MA USA

**Keywords:** Aragonite, Scaffold, Cartilage, Condyles, Trochlea, Microfracture, Knee

## Abstract

**Purpose:**

There is limited comparative evidence on patient outcomes following cartilage repair in various knee compartments. The aim of this study was to compare clinical and imaging outcomes after treating cartilage defects in femoral condyles and trochlea with either an aragonite-based scaffold or surgical standard of care (SSoC, i.e., debridement/microfractures) in a large multicentre randomized controlled trial.

**Methods:**

247 patients with up to three knee joint surface lesions (ICRS grade IIIa or above) in the femoral condyles, trochlea or both (“mixed”), were enrolled and randomized to surgery with either a cell-free aragonite scaffold or SSoC. Patients were followed for up to 48 months by analysing subjective scores (KOOS and IKDC), radiological outcomes (defect filling on MRI), as well as treatment failure rates and adverse events. A differential analysis of outcomes for condylar, trochlear and mixed lesions was performed.

**Results:**

The scaffold group significantly outperformed the SSoC group regardless of lesion location with statistically significantly better KOOS Overall scores at 24 months (all *p* ≤ 0.0009) and 48 months (all *p* ≤ 0.02). Similar results were observed for KOOS subscales and IKDC scores. For KOOS responder rates, superiority of the implant group was demonstrated at 24, 36, and 48 months (all *p* ≤ 0.004). Higher defect filling on MRI for implants was observed for all locations. Lower treatment failure rates for the implant were observed in condylar and mixed lesions.

**Conclusion:**

The aragonite-based scaffold was safe and effective regardless of the defect location, providing superior clinical and radiological outcomes compared to SSoC up to four years follow-up.

**Level of evidence:**

I – Randomized controlled trial.

## Introduction

Cartilage lesions are common causes of knee pain that can impair quality of life as much as end-stage knee osteoarthritis (OA) [1–3]. Clinical impact and treatability of cartilage lesions differs by defect location. The biomechanical and anatomical characteristics of the knee joint exert different forces on cartilage in weightbearing and non-weightbearing areas [4, 5]. The presence of joint contact areas and increased forces has been well studied both in condyles and the trochlea. Knee cartilage defects occur more commonly in condylar areas, where the weightbearing load and joint contact pressure can cause a biomechanically unfavourable environment for cartilage repair [6, 7]. Lesions in the trochlea are generally caused by mechanical problems of the patellofemoral joint and can predispose patients to development of OA if left untreated [8, 9]. Investigations with microfracture and autologous chondrocyte implantation (ACI) have shown that lesion location has a significant impact on clinical outcomes for cartilage restoration procedures. Microfractures tend to provide better results in femoral condyles and tibial plateau defects compared to trochlear and patellar ones [10]. ACI seems to offer limited repair in patellar defects whereas results are quite satisfactory in femoral condyle and trochlear lesions [11]. Few studies have investigated site-based differences in clinical outcomes for biomimetic cell-free scaffolds, which present a single-step approach to treat knee chondral and osteochondral defects [12–14].

An innovative aragonite-based cell-free biomimetic scaffold has shown promising results in subchondral bone and cartilage regeneration with significant clinical improvement up to mid-term evaluation [15–17] A previous publication reporting data at two years follow-up demonstrated the excellent safety profile of the aragonite-based scaffold and its superior efficacy over the current standard of care, i.e., microfractures or debridement in patients with knee chondral/osteochondral defects in the presence of osteoarthritis [15]. No study to date has reported on the effectiveness of an aragonite-based biomimetic cell-free scaffold used in the trochlea as compared to the results in the femoral condyles. The aim of the present study is to provide a differential analysis of the results from a large, multicentre randomized controlled trial (RCT) which compared a novel cell-free aragonite-based scaffold to surgical standard of care (SSoC) for the treatment of chondral/osteochondral lesions in the femoral trochlea and condyles.

## Materials and methods

### Study design

Twenty-six global orthopaedic centres were involved in the RCT. The study was registered on clinicaltrials.gov (NCT: 03299959), approved by the United States of America’s Food and Drug Administration and each site’s Ethics Committees/Institutional Review Boards and was performed in line with the principles of the Declaration of Helsinki. All patients provided written informed consent. Patients were randomized to the aragonite-based scaffold (implant group) or SSoC (control group) in a 2:1 ratio (two patients in implant group vs. one patient in control group). Subjects were assigned to their treatment arm at each study site and within strata defined on the basis of osteoarthritis grade, according to the Kellgren–Lawrence (KL) classification. Details on patient selection and treatment allocation have been previously reported [15]. Enrolment was conducted from September 2017 to November 2019.

### Study device

The Agili-C™ implant (Cartiheal Ltd, Israel), is a biphasic, cell-free implant made of natural calcium carbonate (aragonite) derived from purified, inorganic coral exoskeleton. This biomaterial offers a three-dimensional structure with high interconnected macro-porosity as well as robust mechanical properties, both crucial for the ingrowth of new bone and cartilage tissue. Previous studies have provided a detailed description of the implant’s physicochemical characteristics [18–20].

### Inclusion/exclusion criteria

Main inclusion criteria were: (1) patients aged 21–75 years; (2) presence of up to three joint surface lesion(s), International Cartilage Repair Society (ICRS) Grade IIIa or above; (3) total treatable area 1–7 cm^2^; (4) patients willing and able to comply with post-operative rehabilitation protocol and follow-up schedule.

Main exclusion criteria were: (1) Baseline Knee Injury and Osteoarthritis Outcome Score (KOOS) Pain Subscale score < 20 or > 65 (maximum pain = 0, pain free = 100); (2) bony defect deeper than 8 mm on baseline imaging; (3) articular cartilage lesions in the tibia or the patella, ICRS grade IVa or above; (4) severe osteoarthritis of the index knee, KL Grade 4; (5) instability of the index knee, International Knee Documentation Committee (IKDC), Grade C (abnormal) or D (severely abnormal); (6) Malalignment more than 8 degrees varus or 8 degrees valgus; (7) Lack of functional remaining meniscus, at least 5 mm rim; (8) History of intraarticular or osseous infection of the index knee; (9) uncontained lesion, lack of vital bone wall surrounding the lesion of at least 2 mm thickness.

### Surgical treatments

The aragonite-based scaffold implantation technique has been described in previous publications [15, 16]. Briefly, the surgical site was prepared using an open or mini-open approach by sequentially drilling through the articular surface and into the subchondral bone with a specific tool set. The implant was inserted using a press-fit method ensuring that the implant’s top was flush with the subchondral bone, typically 2 mm or more recessed below the cartilage surface. In cases requiring multiple implants, a minimum 5 mm bone bridge was left to prevent any overlap between biological zones of repair. Implant stability was examined by repeatedly bending the knee before and after removal of the tourniquet. The control group was treated arthroscopically with debridement or microfracture. The appropriate SSoC comparator for each patient (i.e., microfracture vs. debridement) was determined using a previously described algorithm based on patient age, concurrent level of OA, and lesion size [15]. Debridement consisted of removing damaged and unstable cartilage fragments from the articular surface. Microfracture was performed according to the established technique [21].

### Rehabilitation protocol

The rehabilitation program was the same for all study participants and included partial weight bearing for four weeks followed by progressive weight bearing to reach full weight bearing and full range of motion at eight weeks after surgery [15].

### Outcomes

Patients were assessed for up to 48 months after surgery. All adverse events were recorded. The primary endpoint was the change in average KOOS Overall Score from baseline to last follow-up. Secondary outcomes included the KOOS subscales and IKDC Subjective Knee Evaluation score. Treatment failures were defined as any secondary intervention in the index knee, including intra-articular injection or surgery.

All patients underwent MRI at 12 and 24 months to assess the percentage of articular defect fill after surgery. The following MRI protocol was adopted: Field of view: 14 cm; slice thickness 3–3.5 mm; matrix 512 × 256 (or 384); Receiver bandwidth: 80-120Hx/pixel. Sequences: (a) Coronal IW FSE no fatsat; TR ≥ 3000ms; TE = 30-40ms; (b) Coronal PDW FSE with fatsat; TR ≥ 3000ms; TE = 10-20ms; (c) Sagittal IW FSE no fatsat; TR ≥ 3000ms; TE = 30-40ms; (d) Sagittal PDW FSE with fatsat; TR ≥ 3000ms; TE = 10-20ms; (e) Axial IW FSE no fatsat; TR ≥ 3000ms; TE = 30-40ms; (f) Axial T2W FSE with fatsat; TR ≥ 3000ms; TE = ≥ 70ms; (g) Sagittal T1W no fatsat; TR = 600–800; TE = 10-20ms; (h) Oblique PDW FSE with fatsat; TR ≥ 3000ms; TE = 10-20ms oriented perpendicularly to the scaffold. Independent, blinded radiologists with expertise in cartilage repair performed the defect fill assessments. Assessment included at least two sagittal slices and two coronal slices located within the implant/lesion. In the notch and trochlea lesions, axial cuts were used. Cartilage defect volume fill was semi-quantitatively assessed for each slice in increments of 25% fill (i.e.: 0–24%, 25–49%, 50–74% and 75–100%) and results of the slices were averaged. Thus, each score was a composite average of 4–6 slices in both sagittal and coronal orientation. For multiple defects, a single value was calculated by averaging all defects.

### Statistical analysis

The sample size was determined using an adaptive design that employed Bayesian predictive probability in a “Goldilocks” strategy, as detailed in a previous paper [15]. Baseline observation carried forward was applied for patients considered treatment failures. Missing data for reasons other than treatment failure were assumed missing at random and imputed using a maximum likelihood estimation of a mixed model for repeated measures (MMRM). The MMRM included treatment group, baseline values and all available intermediate values of changes over time. This approach produces unbiased estimates of the treatment effect under the assumption that, conditional on these variables, the likelihood of missing data is independent of the distribution of the missing values. The MMRM was used to determine the 48-month treatment group contrast, 95% confidence intervals, and *p*-values. Baseline characteristics and outcomes were summarized using descriptive statistics. Patients were categorized into the condylar, trochlear, and mixed-lesions groups based on defect location. The mixed-lesions group consisted of patients with concomitant condylar and trochlear cartilage lesions. Outcomes for this group were compared to the “isolated” condylar and trochlear lesion groups to assess potential of the implant in complex cases. For all tests *p* < 0.05 was considered statistically significant.

## Results

### Patients demographics

Isolated defects in the condyles were seen in 137 patients, *n* = 86 (52.4%) for the implant group and *n* = 51 (61.4%) for SSoC. Lesions were most commonly located in the medial femoral condyle (*n* = 54 in the implant group and *n* = 36 in the SSoC group), and less frequently in the lateral femoral condyle (*n* = 25 in the implant group and *n* = 19 in the SSoC group) or in both condyles (*n* = 7 in the implant group and *n* = 5 in the SSoC group).

A total of 48 patients had isolated defects in the femoral trochlea, *n* = 34 (20.7%) for the implant group and *n* = 14 (16.9%) for SSoC. The ‘mixed-lesions’ group consisted of 62 patients, n = 44 (26.8%) for the implant group and n = 18 (21.7%) for SSoC. Demographic characteristics of these subgroups are summarized in Table 1. There were no statistically significant differences between groups, with the exception of younger age and lower BMI in the scaffold group with condylar lesions, however those differences were considered clinically insignificant. There were no differences between groups in the baseline KOOS Overall, KOOS Subscale, and IKDC scores.

**Table 1:** Summary of baseline demographics by lesion location

	Agili-C™			SSoC			Agili-C™ - SSoC		
**Isolated Trochlear Defects**	***N***	**Mean**	**SD**	***N***	**Mean**	**SD**	**Diff**	**LB**	**UB**
Age	34	40.4	9.6	14	41.9	9.2	-1.46	-7.52	4.60
Height (cm)	34	177.8	7.7	14	176.6	8.1	1.15	-3.86	6.17
Weight (kg)	34	83.1	15.0	14	85.8	13.7	-2.74	-12.11	6.62
BMI (kg/m^2^)	34	26.2	4.0	14	27.5	4.2	-1.30	-3.89	1.29
**Isolated Condylar Defects**	**N**	**Mean**	**SD**	**N**	**Mean**	**SD**	**Diff**	**LB**	**UB**
Age	86	39.7	11.2	51	46.2	11.6	-6.46	-10.42	-2.50
Height (cm)	86	173.4	9.2	51	173.2	9.8	0.19	-3.10	3.47
Weight (kg)	86	76.7	15.6	51	83.7	14.5	-6.94	-12.25	-1.63
BMI (kg/m^2^)	86	25.4	3.9	51	27.8	3.9	-2.44	-3.80	-1.08
**Multicompartmental Defects (Mixed Lesions)**	**N**	**Mean**	**SD**	**N**	**Mean**	**SD**	**Diff**	**LB**	**UB**
Age	44	47.0	10.2	18	48.4	10.0	-1.45	-7.14	4.24
Height (cm)	44	176.1	8.9	18	175.6	11.5	0.47	-4.97	5.91
Weight (kg)	44	88.8	15.2	18	88.1	16.5	0.71	-7.98	9.41
BMI (kg/m^2^)	44	28.6	4.2	18	28.4	3.7	0.19	-2.06	2.45

*SSoC: Surgical Standard of Care; N: number*,* SD: standard deviation*,* Diff: difference*,* LB: lower bound*,* UB: upper bound*

### Patient reported outcomes

**Fig. 1:**
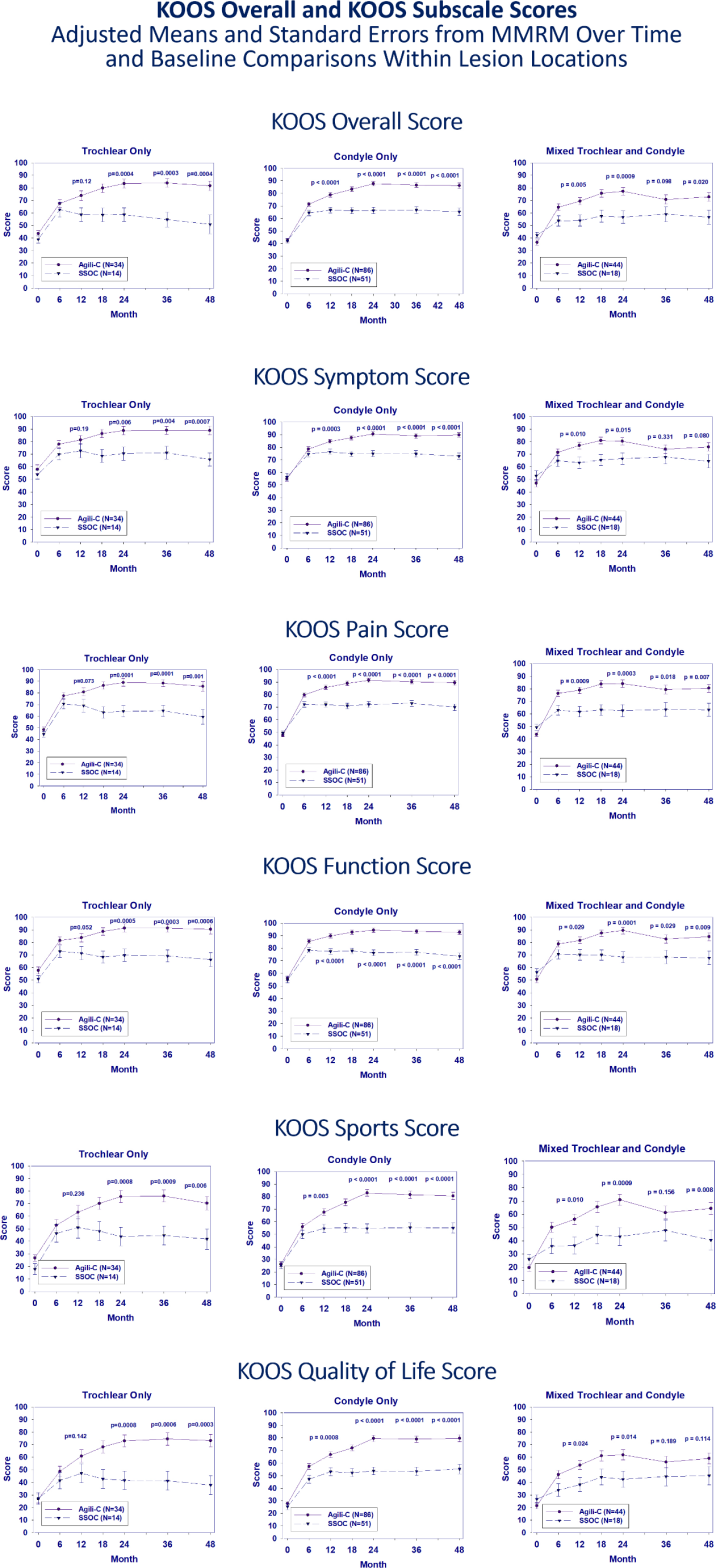
Change from Baseline to the 48 months follow up in KOOS Overall Score and all Subscales for each lesion location comparing Agili-C to the Surgical Standard of Care (SSoC)

**Fig. 2:**
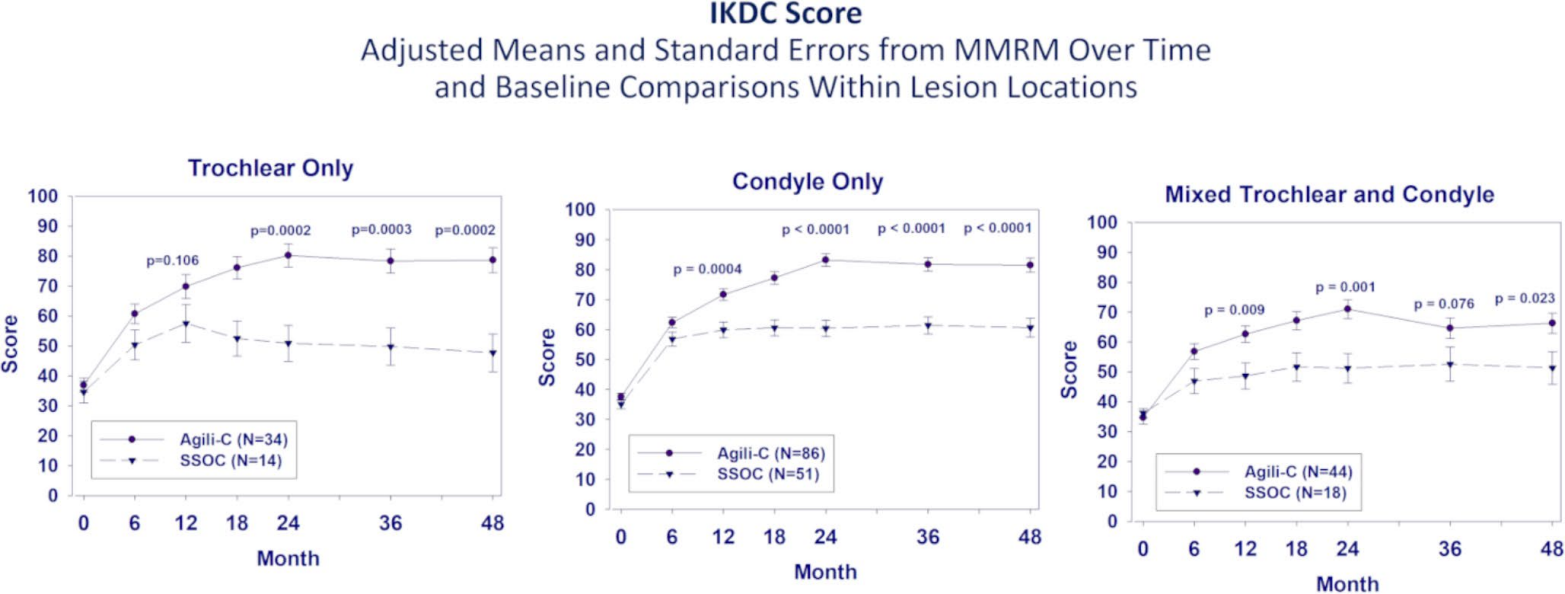
Change from baseline to the 48 months follow up in IKDC Score for each lesion location comparing Agili-C to the Surgical Standard of Care (SSoC)

The KOOS Overall score in the condylar and mixed-lesions subgroups was significantly higher for the implant group compared to controls at 12 months (*p* ≤ 0.005). The trochlear group also demonstrated better clinical scores at the 12 months follow up but statistical significance was reached only at 24 months of follow up. All three site-specific groups treated with the implant had significantly higher KOOS Overall score in all subsequent timepoints. By 48 months, superiority margins were larger and statistically significantly higher for all lesion location subgroups, showing a mean difference of 20.8 in condylar defects (*p* < 0.0001), 27.0 in trochlear defects (*p* = 0.0004) and 16.1 in mixed lesions (*p* = 0.0198). Similar trends were observed for all KOOS subscales (Fig. 1) and the IKDC subjective score (Fig. 2) in all location subgroups. Scores for the implant group were significantly higher at 12 months with continued increases to 24 months and stable scores through 48 months. Mixed-lesion patients in both groups, implant and SSoC, had overall lower clinical scores than those with isolated condylar or trochlear defects. Furthermore, most patients were able to regain full active range of motion after approximately two months.

### Responder rate

**Fig. 3:**
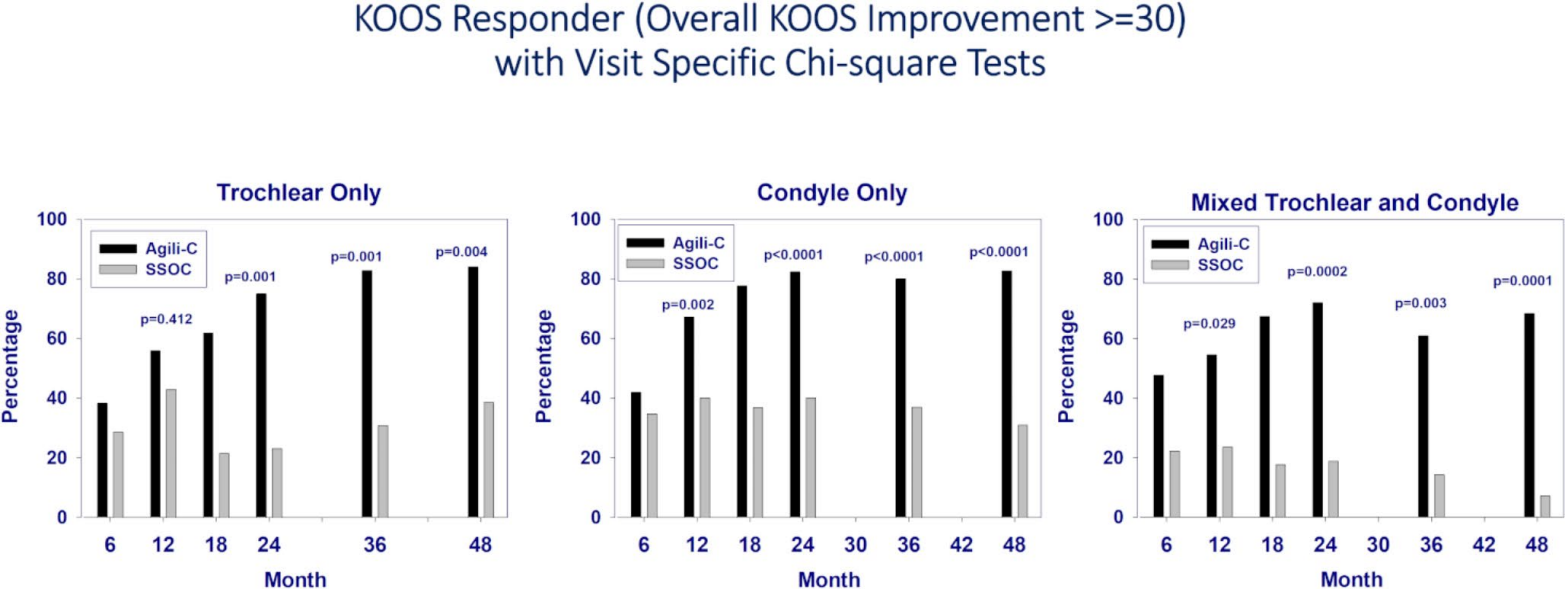
Change from baseline to the 48 months follow up in the percentage of responders (intended as those obtaining > 30 improvement in KOOS Overall Score) for each lesion location comparing Agili-C to the Surgical Standard of Care (SSoC)

The implant group outperformed controls in responder rate in all the location subgroups. At 48 months the responder rate in the implant group reached 84.0% in trochlear defects (vs. 38.5% in controls, *p* = 0.004), 82.7% in condylar defects (vs. 31.0% in controls, *p* < 0.0001), and 68.4% in mixed-lesions (vs. 7.1% in controls, *p* = 0.0001). (Fig. 3).

### Imaging outcomes

Defect filling of ≥ 75% was used as the threshold to identify patients with satisfactory MRI outcomes. Significantly superior imaging outcomes were seen in the scaffold group compared to the control group at the 12- and 24-month evaluations (Table 2). For condylar defects 93.9% of patients in the scaffold group achieved at least 75% defect fill at 24 months compared to 39.0% of patients in the control group (*p* < 0.0001). In trochlear defects, 62.5% of patients in the scaffold group achieved at least 75% defect fill compared to 18.2% of controls (*p* = 0.012). In the mixed-lesions group, 97.6% of scaffold patients had at least 75% defect fill at 24 months compared to 18.8% of control patients (*p* < 0.0001).

**Table 2:** Summary of MRI defect fill at months 12 and 24 by Lesion Location

Isolated Trochlear Defects						Isolated Condylar Defects					Multicompartmental Defects (Mixed Lesions)						
	Agili-C^1^		SSOC			Agili-C^1^		SSOC			Agili-C^1^		SSOC				
**Month 12 MRI Defect Fill (%)**																	
	**n**	**%**	**n**	**%**	**p-value** ^**2**^	**n**	**%**	**n**	**%**	**p-value** ^**2**^	**n**	**%**		**n**	**%**		**p-value** ^**2**^
>=75%	24	70.6	3	21.4	0.0020	73	89.0	20	43.5	0.0000	41	97.6		3	17.6		0.0000
< 75%	10	29.4	11	78.6		9	11.0	26	56.5		1	2.4		14	82.4		
**Month 24 MRI Defect Fill (%)**																	
	**n**	**%**	**n**	**%**	**p-value** ^**2**^	**n**	**%**	**n**	**%**	**p-value** ^**2**^	**n**	**%**		**n**	**%**		**p-value** ^**2**^
>=75%	20	62.5	2	18.2	0.0122	77	93.9	16	39.0	0.0000	41	97.6		3	18.8		0.0000
< 75%	12	37.5	9	81.8		5	6.1	25	61.0		1	2.4		13	81.3		

**Notes**:

^1^ Agili-C: aragonite-based scaffold, SSoC: Surgical Stardard of Care

^2^*P*-value for Wilcoxon rank sum test

### Influence of OA and lesion size

Patients were stratified according to Kellgren-Lawrence (KL) grade, comparing those with no or minimal OA (KL grade 0 or 1) to those with mild to moderate OA (KL grade 2 or 3). Absence or presence of osteoarthritis did not impact the outcomes of the aragonite scaffold, which provided significant and comparable improvement in condylar, trochlear and mixed-lesions subgroups at the 48-month evaluation. The stratification by lesion size (≤ 3 cm^2^ or > 3 cm^2^) showed no significant influence on clinical outcomes for the scaffold group; KOOS overall scores in both lesion size strata were higher than the SSoC group across all lesions locations. For all three lesion location subgroups, the superiority margin was numerically larger among patients with total lesion(s) size > 3 cm^2^. This difference was statistically significant for patients with condylar lesions, where the superiority margin was 28.8 (*p* < 0.0001) and 14.0 (*p* = 0.004) among patients with total lesion(s) size > 3 cm^2^ vs. ≤ 3 cm^2^, respectively. The lesion size by treatment group interaction was statistically significant (*p* = 0.045). Moreover, in patients with implant treated condylar defects, comparable clinical and radiological outcomes were seen irrespective of the defect location (medial vs. lateral vs. both condyles).

### Treatment failures and adverse events

At four-year follow-up, lower treatment failure rates for the implant compared to SSoC were observed for condylar (*p* = 0.001) and mixed lesions (*p* ≤ 0.017). Failure rate was also lower, although not significantly, among implanted patients with trochlear lesions (*p* = 0.099). The failure rate for the scaffold group was 8.1% in condylar defects, 11.8% in trochlear defects, and 13.6% in mixed lesions. The control group showed failure rates of 31.4% in condylar defects, 35.7% in trochlear defects and 44.4% in mixed lesions. The control group experienced a significantly higher rate of “transient or persisting pain” compared to the scaffold group in both condylar and trochlear defects. There were no differences in the rate of adverse events between location subgroups. At the 48-month follow-up, 1.2% (*n* = 2) of patients in the scaffold group and 9.5% (*n* = 8) of patients in the control group had undergone a knee replacement or osteotomy (*p* = 0.003).

## Discussion

The main finding of this randomized controlled trial is that the aragonite-based scaffold provides better clinical outcomes than debridement/microfractures at mid-term follow-up for condylar and trochlear cartilage defects. Clinical results showed the clinical superiority of the scaffold for treating condylar and trochlear lesions for all lesion sizes and grades of OA. Mixed-lesion patients in both groups had lower clinical scores than those with isolated defects, as expected due to the intrinsic higher complexity of cases with defects in multiple locations. The implant was superior to SSoC for these complex cases as evidenced by the significantly higher clinical and imaging outcomes for this group. The study was powered to demonstrate differences between the implant and SSoC pooling over all lesion locations. The finding that the implant remained statistically significantly superior to SSoC within each of the lesion location reflects the very large superiority margins observed in all subgroups.

Osteochondral repair has presented persistent challenges for orthopaedic surgeons [22]. First attempts with osteochondral autografts showed the right therapeutic direction, although limited by donor site morbidity and deteriorating long-term outcomes [23–25]. Advancements in the field of biomaterials paved the way for one-step surgical procedures for full-thickness cartilage defects [26]. The specific location of the cartilage lesion deserves special attention when choosing the best therapeutic solution for a patient. Patellofemoral lesions are frequently considered an intrinsically distinct condition from condylar lesions given the peculiar anatomy and biomechanics of the patellofemoral joint [27]. The morphology of the patella and the trochlea can have wide variability between patients and is associated with pathologic mal-tracking, malalignment and dysplasia that can further increase joint reaction forces that are already significantly higher than those in the tibiofemoral joint [4, 28, 29]. There have been very few studies comparing the clinical results of cartilage restoration procedures based on the specific location.

A recent systematic review by van Tuijn et al. analysed the different factors influencing the clinical outcomes of microfracture, the most common cartilage procedure [30]. The group found that the influence of lesion location had only been investigated by seven studies in the present literature and concluded that a favourable outcome after microfracture could only be predicted when dealing with single, non-degenerative, non-weightbearing lesions not involving the femoral trochlea or patella [30]. On the other hand, autologous chondrocyte implantation (ACI), either matrix-assisted (MACI) or not, has been shown to produce a smaller clinical benefit for condylar defects than for patellar or trochlear defects [31–33]. Osteochondral allograft transplantation (OCA) is particularly challenging for lesions of the femoral trochlea because its unique anatomy presents significant challenges in matching suitable donors to patients [34]. Only one study has examined the impact of lesion location for biomimetic cell-free scaffolds; in their cohort of 79 patients treated with the implantation of a blended hydroxyapatite/collagen biomimetic scaffold, Kon et al. found that the lesion location (either medial femoral condyle, lateral femoral condyle, or femoral trochlea) was not correlated with clinical outcomes [12].

In the present analysis, we were able to demonstrate that the aragonite-based scaffold provides comparable beneficial effects in isolated condylar, isolated trochlear and multicompartmental cartilage defects with up to four years’ follow-up, regardless of lesion size and concurrent presence of OA. This highlights the versatility of the present scaffold, which can be successfully used in the “complex” patients who are routinely seen in clinical practice, such as those with multiple lesions, large defects, and signs of degenerative joint changes. Patients with patellofemoral cartilage damage are particularly challenging to manage; in the present series the aragonite scaffold revealed very satisfactory results maintained up to middle term for patients affected by high grade trochlear lesions with concurrent minor patellar cartilage damage. Even patients with concurrent condylar and trochlear defects reported significant benefit following scaffold implantation, although with lower responders’ rates and clinical scores compared to patients with isolated lesions.

The rehabilitation protocol plays a significant role in recovery after chondral or osteochondral procedures. Determining the appropriate rehabilitation protocol for a particular patient or procedure is often a source of debate among orthopaedic surgeons [35, 36]. Some authors advocate the necessity of different protocols for different defect locations, especially when the patellofemoral compartment is involved [37]. In our study, the same rehabilitation protocol was adopted for both condylar and trochlear patients, thus avoiding a potential bias in outcome evaluation.

Another major strength of the present study is the strict patient selection process, whereby only patients who did not need major concurrent procedures (such as patellar stabilization, ligament reconstruction, meniscal allograft transplantation) were included, thus enabling the assessment of the true clinical potential of the scaffold in regenerating the osteochondral unit without the presence of other confounding factors.

Nonetheless, we must acknowledge some limitations. Randomization in the location subgroups was unequal because enrolment was not stratified by lesion location and because isolated condylar lesions are much more common than isolated trochlear lesions. Further, the control group received either debridement or microfractures, which are distinct procedures that can provide different clinical performance. Patients in the SSoC arm were allocated to microfracture vs. debridement using an algorithm based on patient age, concurrent presence of OA and lesion size described in a previous publication [15]. This design was selected because of the different clinical indications for debridement and microfracture. Poolability and sensitivity analyses were conducted to ensure the statistical appropriateness of combining into a single control group, but the study was not designed to be able to compare effectiveness of debridement vs. microfracture because these were applied in different patient populations. Another limitation is that the MRI assessment of the defect fill performed with a semi-quantitative analysis (25% increments) rather than a fully quantitative scoring method as used previously to evaluate cartilage repair with autologous chondrocyte transplantation.

## Conclusion

The aragonite-based scaffold provided superior clinical improvement compared to debridement/microfractures up to four years’ follow-up in patients affected by isolated condylar or trochlear defects. Complex patients with concurrent trochlear and condylar (mixed) defects treated with the scaffold showed significantly superior clinical improvement compared to the control group. The scaffold’s clinical performance was not affected by lesion size or degree of concurrent OA.
